# Optic nerve sheath meningioma exhibits neural niche‐associated transcriptomic features and rare copy number variation‐linked evolution

**DOI:** 10.1111/bpa.70078

**Published:** 2026-02-01

**Authors:** Daisuke Sato, Satoru Miyawaki, Yu Sakai, Kenta Ohara, Yu Teranishi, Yudai Hirano, Motoyuki Umekawa, Takahiro Tsuchiya, Shotaro Ogawa, So Hirata, Hiroki Hongo, Hideaki Ono, Daisuke Komura, Tetsuo Ushiku, Shumpei Ishikawa, Nobuhito Saito

**Affiliations:** ^1^ Department of Neurosurgery The University of Tokyo Tokyo Japan; ^2^ Department of Neurosurgery Tokyo Metropolitan Geriatric Hospital and Institute of Gerontology Tokyo Japan; ^3^ Department of Preventive Medicine, Graduate School of Medicine The University of Tokyo Tokyo Japan; ^4^ Department of Pathology, Graduate School of Medicine The University of Tokyo Tokyo Japan

**Keywords:** copy number alteration, copy number variation, meningioma, optic nerve sheath meningioma, transcriptomic

## Abstract

Optic nerve sheath meningioma (ONSM) is a rare tumor that arises from the meninges enveloping the optic nerve. Although the genetic landscape of meningiomas has been extensively studied, the molecular alterations underlying ONSM remain poorly understood. We retrospectively analyzed consecutive patients surgically treated for ONSM between 2000 and 2025 at our institution, with available histological specimens. Intracranial meningiomas secondarily extending into the optic canal were excluded. Fresh‐frozen tumor samples were subjected to whole‐exome sequencing, and transcriptomic analyses were conducted and compared with those of intracranial meningiomas from four public datasets. Six cases were included, five of whom were female, with a median age of 63.5 years. While most cases remained stable after surgery, one patient experienced multiple recurrences and ultimately succumbed. Primary tumors were characterized by the absence of *NF2* alterations, occasional *POLR2A* mutations, and few copy number variations (CNVs). Transcriptomic profiling in primary tumors revealed a neurotrophic microenvironment reflective of the close association with the optic nerve. The recurrent case exhibited high‐risk CNVs at diagnosis and developed into an aggressive disease as additional CNV burdens accumulated, including the homozygous deletion of *CDKN2A/B*. Its expression profile was in line with that of hypermitotic, proliferative intracranial meningiomas. ONSM represents a predominantly *NF2*‐intact meningioma subtype defined by neural niche‐associated transcriptional signatures. Although typically indolent, ONSM can, in rare instances, evolve into an aggressive disease through further accumulation of CNVs.

## Graphical abstract

Optic nerve sheath meningiomas are typically *NF2*‐intact with few copy number alterations and are generally clinically indolent. Rare aggressive recurrences are associated with progressive accumulation of copy number variations, including *CDKN2A/B* homozygous deletion, 1q gain, and 14q loss.

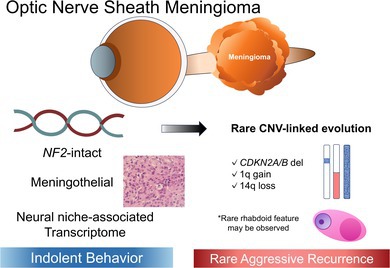

## 1 INTRODUCTION

Meningiomas represent the most common primary intracranial tumors, accounting for 25%–40% of all cases [1, 2]. Optic nerve sheath meningioma (ONSM) is rare, comprising approximately 2% of all meningiomas and about 2% of intraorbital tumors [3, 4]. As meningiomas generally arise from the meninges, ONSM originates from the meninges covering the optic nerve [4]. These tumors typically grow slowly and may cause progressive visual impairment because of direct compression of the optic nerve or compromise of the pial vasculature [5, 6]. Other clinical manifestations of ONSM include proptosis, optic disc edema or atrophy, and optociliary shunt vessels [6].

While landmark studies have advanced our understanding of the molecular landscape of meningiomas [7, 8, 9, 10, 11, 12, 13, 14, 15, 16, 17], the molecular basis of ONSM remains poorly characterized. To date, only limited studies have reported ONSM cases associated with neurofibromatosis type 2 [18, 19] or postzygotic somatic mosaicism of *TRAF7* [20], along with a single‐nucleotide polymorphism‐based array analysis showing 22q loss in one of five cases (20%) [21]. Although these findings suggest that ONSM may exhibit non‐random genetic features, its molecular underpinnings remain largely unknown.

We consider ONSM to be unique because, unlike in other intracranial regions, the derivative meninges intimately envelop the cranial nerve [22], creating a distinctive anatomical interface that may underlie its singular biology. From a nomenclatural standpoint as well, ONSM stands alone among meningiomas as the only subtype named after a cranial nerve, further underscoring its uniqueness. To investigate the molecular basis of this exceptional entity, we performed genomic and transcriptomic analyses of institutional ONSM cases. While the mortality rate of ONSM has been reported to be virtually zero [3, 6, 23, 24], a recent report describing a rhabdoid variant has raised concern for potential aggressive behavior [25]. In light of these observations, we encountered a case with multiple recurrences and distant metastasis that ultimately resulted in tumor‐related death. Here, we delineate the molecular landscape of ONSM and highlight the potential genetic disruptions underlying rare tumor evolution.

## 2 MATERIALS AND METHODS

### 2.1 Sample collection and data acquisition

We included consecutive patients who underwent surgical resection for ONSM and had available histological tissue at our institution between January 1, 2000, and July 31, 2025. Meningiomas originating from the intracranial region and extending into the optic nerve were excluded. One case (*Case 1*) was included in our previous multi‐omics study, which analyzed paired pre‐ and post‐recurrence meningioma specimens to delineate molecular alterations associated with malignant transformation [26]. For comparative analyses, publicly available transcriptomic datasets were utilized (GSE136661 [13], GSE183653 [10], GSE252291 [17], and GSE270638 [16]).

The study was approved by the Institutional Ethics Committee (G10028), and written informed consent was obtained from all participants.

### 2.2 Whole‐exome sequencing

Genomic DNA was isolated from snap‐frozen tumor specimens using the QIAamp DNA Mini Kit (Qiagen, Venlo, The Netherlands), and its purity and concentration were verified spectrophotometrically. Sequencing libraries were prepared from acoustically sheared DNA fragments (Covaris, Woburn, MA, USA) generated for whole‐exome analysis with the Twist Comprehensive Exome Panel (Twist Bioscience, San Francisco, CA, USA). Following adapter ligation, exonic targets were enriched through hybrid capture with biotin‐labeled RNA baits and retrieved using streptavidin‐coated magnetic beads. The captured material underwent polymerase chain reaction (PCR) amplification and was subsequently sequenced on an Illumina NovaSeq 6000 platform with 150‐bp paired‐end reads.

Raw reads were processed using fastp for adapter trimming and quality filtering, and alignment to the human reference genome (GRCh38) was performed with the Burrows–Wheeler Aligner. Somatic variants were called in tumor‐only mode using Mutect2 (Parabricks v3.8.0) and filtered with FilterMutectCalls (GATK v4.1.7.0) to reduce potential artifacts. Copy number profiles were inferred with CNVkit (v0.9.10), referencing pooled normal blood DNA from five individuals.

Subsequent analyses focused on driver alterations and clinically relevant genomic events, including *TERT* promoter mutations and homozygous deletions of *CDKN2A/B*, both recognized in the 2021 World Health Organization (WHO) classification of meningiomas. Large‐scale arm‐level copy number variations (CNVs) were defined as regions covering >80% of a chromosome arm, and *CDKN2A/B* homozygous deletion was characterized by a log_2_ copy ratio below −1.1 across the locus and its neighboring regions [27].

### 2.3 Specimen processing for bulk RNA sequencing

Total RNA was extracted from snap‐frozen tumor samples using the RNeasy Mini Kit in combination with the RNase‐Free DNase Set (Qiagen, Hilden, Germany). RNA integrity was verified on an Agilent 2100 Bioanalyzer (Agilent Technologies, Santa Clara, CA, USA), and RNA concentration was determined with a NanoDrop spectrophotometer (Thermo Fisher Scientific, Waltham, MA, USA). Polyadenylated RNA was isolated from total RNA using oligo(dT)‐conjugated magnetic beads and subsequently fragmented by heat treatment. Complementary DNA (cDNA) was synthesized using SuperScript II reverse transcriptase (Invitrogen, Carlsbad, CA, USA), followed by adapter ligation and PCR amplification with the TruSeq Stranded mRNA Library Prep Kit (Illumina, San Diego, CA, USA). For one sample with insufficient input RNA concentration (*Case 6*), cDNA synthesis and amplification were performed using the SMART‐Seq Stranded Kit (Takara Bio USA, San Jose, CA, USA), followed by Nextera XT tagmentation for library construction. Constructed libraries were sequenced on an Illumina NovaSeqX Plus platform to generate 150‐bp paired‐end reads.

Sequencing reads were aligned to the human reference genome (GRCh38/hg38) using STAR (v2.7.10), gene‐level read counts were summarized with FeatureCounts (v2.0.6), and transcript abundance was quantified in transcripts per million (TPM) using StringTie (v2.1.7).

Public RNA sequencing datasets were processed using the same alignment and quantification pipeline as for the institutional specimens described above.

### 2.4 RNA sequencing analyses and visualization

Raw gene counts from each dataset were combined, and batch effects were corrected using ComBat‐seq implemented in the R package “sva.” [28] Gene expression values from the combined datasets were normalized using the Variance Stabilizing Transformation method [29]. Principal component analysis (PCA) was performed on all genes, and the resulting components (default parameters for t‐distributed stochastic neighbor embedding (t‐SNE) initialization) were used to generate a t‐SNE visualization with a perplexity of 30 and a maximum of 1000 iterations.

The adult dural signature and cell cycle‐related gene sets were defined as described below, and the Gene Set Variation Analysis (GSVA) method was applied to estimate the transcriptomic activity of each case associated with these gene sets. The adult dural signature gene set was defined by identifying the top 50 differentially expressed genes (DEGs) of adult dural fibroblast using public single‐cell RNA sequencing datasets (GSE183655 and GSE206647), which included the following: *MFAP5*, *SERPINA3*, *PLA2G2A*, *DPT*, *SVEP1*, *LINC01638*, *SFRP2*, *KIAA1755*, *ENPP6*, *FBLN1*, *ZIC2*, *LINC01133*, *SFRP4*, *PTN*, *THBS2*, *NDNF*, *LRRN4CL*, *SLPI*, *SOX9*, *C16ORF89*, *SLC47A1*, *SLC16A9*, *EYA2*, *DCN*, *CHODL*, *ADAMTSL3*, *CRABP1*, *SCARA5*, *SFRP1*, *RBP4*, *CFB*, *THSD4*, *CLMP*, *RFLNA*, *LRP1B*, *SEMA3C*, *KCNMA1*, *LOX*, *MXRA5*, *PDGFRL*, *PLEKHA6*, *FMOD*, *PTHLH*, *ITGBL1*, *CEMIP*, *CRABP2*, *CYP3A5*, *GXYLT2*, *CHRDL1*, and *CDON*. In accordance with previous studies [9, 30], the following genes were defined as cell cycle‐related gene set: *CDC20*, *MYC*, *E2F8*, *CCND1*, *CHEK1*, *CDK1*, *TET1*, *MKI67*, *CDK4*, *TOP2A*, *BRCA1*, *CDKN2A*, *FOXM1*, *TP53*, *MCM2*, *MCM6*, and *MCM4*.

After log‐normalization and batch correction using ComBat, non‐negative matrix factorization (NMF) was performed. Consistent with the approach by Patel et al., we set *k* = 3 and used the top 1500 variable genes for factorization [13]. After 100 iterations, each case was assigned to the cluster with the highest coefficient in the factorized matrix. The resulting coefficients were visualized as a heatmap. The three NMF clusters were subsequently subjected to Gene Ontology Biological Process (GO:BP) analysis, from which the biological characteristics of each cluster were inferred.

Bulk RNA sequencing data were deconvoluted using the BayesPrism package (v 2.2.2) in R [31]. Expression profiles of nine cell types—tumor cells, macrophages, T/natural killer (NK) cells, B cells, endothelial cells, neural cells, mast cells, neutrophils, and pericytes—were derived from public single‐cell RNA sequencing datasets (GSE183655 [10], GSE206647 [32], and GSE213544 [33]) and used as reference matrices for deconvolution. Because of computational constraints associated with large cell numbers, 15,000 tumor cells and 2000 macrophages were randomly subsampled to construct the reference datasets. Other cell types were further subsampled to a total of 500 cells each.

Differential gene expression analysis was performed using the DESeq2 package (v1.42.1) in R [34]. Log_2_ fold changes were shrunk using the apeglm method to stabilize variance estimates and improve visualization [35]. Volcano plots were generated based on the shrunken results. For subsequent gene set enrichment analysis (GSEA), the non‐shrunken differential expression results were used. We performed pre‐ranked GSEA using all genes from the DESeq2 comparison. Genes were ranked by the Wald test statistic in descending order. Analyses were run with the fgsea package (v1.32.4). Adjusted *p*‐values were calculated using the Benjamini–Hochberg method, and terms with an adjusted *p*‐value <0.05 were considered significantly enriched.

To further characterize transcriptional programs intrinsic to ONSM in a complementary, network‐based manner, we additionally performed weighted gene co‐expression network analysis (WGCNA) [36]. Data from the institutional ONSM cohort were integrated with publicly available meningioma datasets, and a signed co‐expression network was constructed using the WGCNA framework. Module eigengenes (MEs), representing the first principal component of each gene module, were calculated and used for downstream analyses. To identify ONSM‐associated transcriptional modules, ME values were compared between the institutional ONSM samples and public meningioma samples. Modules showing characteristic enrichment in the institutional dataset were considered ONSM‐associated and were further interrogated for functional enrichment. This analysis was performed as a complementary approach to GSEA to robustly delineate transcriptional programs associated with ONSM.

## 3 RESULTS

### 3.1 Clinical demographics

**FIGURE 1:**
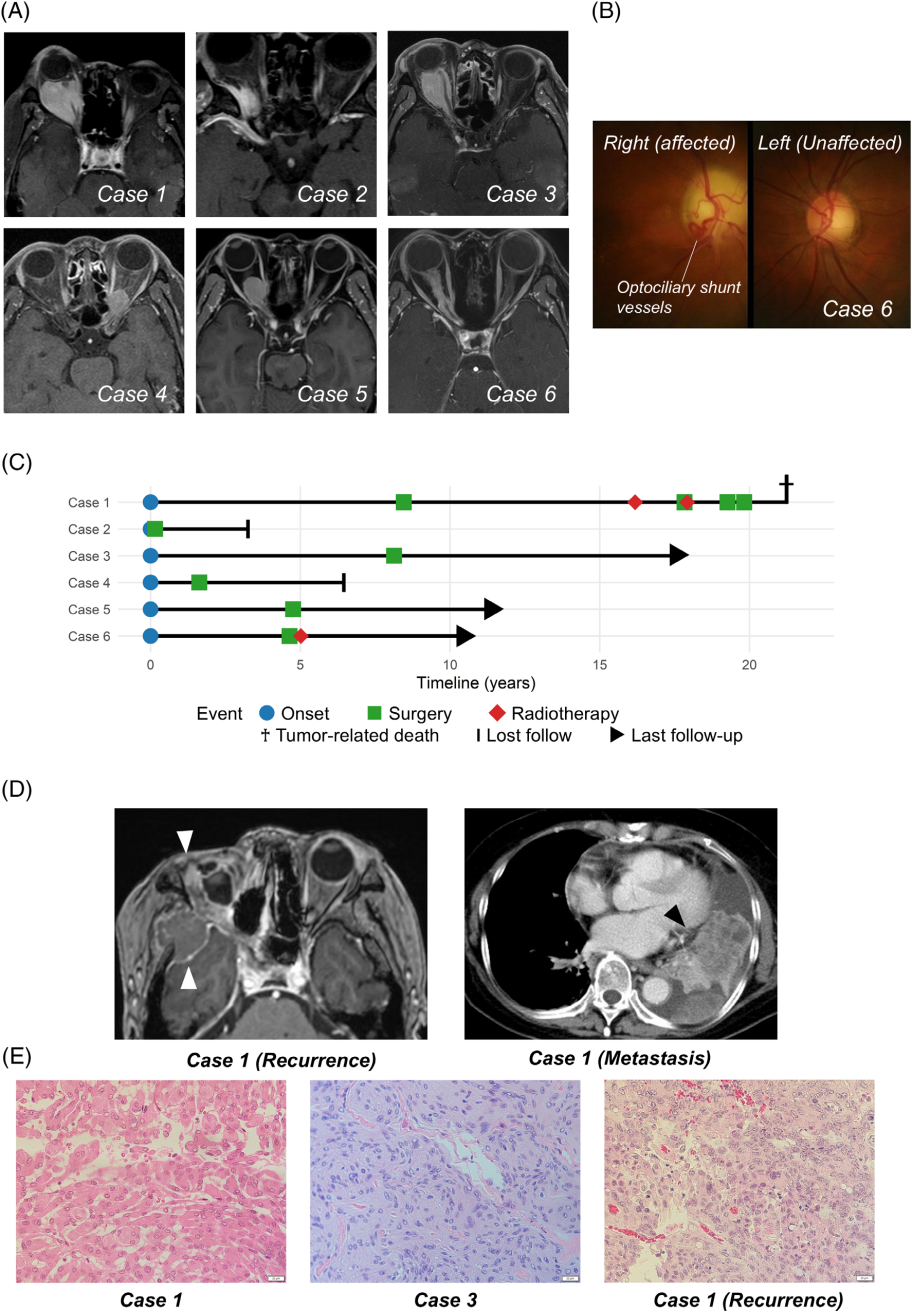
Clinical and histopathological overview. Six cases were diagnosed and treated for optic nerve sheath meningioma during the study period. (A) Representative images are demonstrated for each case. A typical tram‐track sign is observed in *Cases 1*, *3*, and *6*. *Case 2* underwent an open biopsy at another institution, and the image shown was acquired in the interval between the biopsy and the definitive tumor resection, demonstrating post‐biopsy reactive changes. Pre‐biopsy images were unavailable. (B) Fundoscopic examination for *Case 6* reveals a swollen optic disc accompanied by dilated optociliary shunt vessels. (C) The clinical course of all cases is summarized in a time‐course plot. While *Case 1* experienced multiple recurrences, none of the other cases showed recurrence during the follow‐up period. (D) Representative images of recurrence. *Case 1* developed a recurrent tumor with destructive extracranial extension (white arrow head) and pulmonary metastasis (black arrow head) associated with pleural effusion. (E) Histopathological findings of representative cases. All tumors show meningothelial histology at initial presentation. However, *Case 1* already exhibits morphological deviations with partial rhabdoid features, characterized by abundant eosinophilic cytoplasm and eccentrically placed nuclei (left). *Case 3* shows eosinophilic cytoplasm and uniform nuclei with fine chromatin, forming whorled and syncytial structures with intranuclear pseudoinclusions (middle). The recurrent tumor of *Case 1* demonstrates frank anaplasia with focal rhabdoid features (right).

The clinical demographics are summarized in Table 1. Six patients were diagnosed and treated for ONSM during the study period. Five patients (83%) were female, and the median age at diagnosis was 63.5 years (range, 47–82 years). All patients were suffering from visual dysfunction, and two patients had only light perception. Three patients had exophthalmos. Preoperative imaging revealed a tubular expansion in one case, a globular configuration in two cases, a fusiform shape in two cases, and a focal enlargement in one case (Figure 1A). Tram‐track sign was observed in three cases. Figure 1B demonstrates the characteristic optociliary shunt vessels and optic disc swelling observed in *Case 6*.

**TABLE 1:** Clinical demographics.

Case no.	Age/sex	Presentation	Visual function	Laterality	Imaging features	Surgery	Primary tumor histology	Post‐operative radiotherapy	Relapse	FU period (months)	Latest outcome
1	68 years/F	Visual dysfunction, exophthalmos	Counting fingers	R	Fusiform shape	Craniotomy	WHO Grade 1, meningothelial	EBRT 60Gy/30fr	Four episodes	153	Tumor‐related death
2	47 years/F	Visual dysfunction	Moderate impairment	R	Focal enlargement	Craniotomy	WHO Grade 1, meningothelial	‐	‐	44	Stable (moderate visual impairment)
3	73 years/M	Visual dysfunction, exophthalmos	Light perception	R	Fusiform shape	Craniotomy	WHO Grade 1, meningothelial	‐	‐	116	Stable (no light perception)
4	59 years/F	Visual dysfunction	Moderate impairment	L	Globular configuration	Craniotomy	WHO Grade 1, meningothelial	‐	‐	57	Stable (moderate visual impairment)
5	82 years/F	Visual dysfunction	Moderate impairment	R	Globular configuration	Endoscopic trans‐spenoidal	WHO Grade 1, meningothelial	‐	‐	82	Stable (moderate visual impairment)
6	50 years/F	Visual dysfunction	Light perception	R	Tubular expansion	Craniotomy	WHO Grade 1, meningothelial	GKS 15Gy	‐	72	Stable (no light perception)

Abbreviations: EBRT, external beam radiation therapy; F, female; FU, follow‐up; GKS, gamma knife surgery; L, left; M, male; R, right; WHO, World Health Organization.

All patients underwent craniotomy, and the extent of resection was Simpson grade IV in all cases. The optic nerve was resected in *Case 6* but preserved in the other cases. None of the cases underwent preoperative irradiation, and two received post‐operative radiotherapy (*Cases 1* and *6*). The median follow‐up period was 77 months (range, 44–153 months), during which one patient experienced multiple recurrences and ultimately succumbed to the disease (*Case 1*; Figure 1C,D).

All primary tumors were consistent with central nervous system (CNS) WHO grade 1 meningothelial meningiomas. Notably, however, *Case 1* already exhibited atypical histological features at the time of the initial surgery, including focal rhabdoid morphology (Figure 1E). Despite these concerning findings, the tumor did not fulfill additional histological criteria required for upgrading, such as small cells with a high nuclear‐to‐cytoplasmic ratio, prominent nucleoli, sheeting architecture, or foci of spontaneous necrosis. *Case 1* subsequently recurred with extracranial extension and distant metastasis. These recurrent lesions demonstrated frank anaplasia, as shown in Figure 1E, and fulfilled the histomorphological criteria for CNS WHO Grade 3 meningioma. The recurrent tumor also partially retained rhabdoid features.

### 3.2 Genetic profiles

**FIGURE 2:**
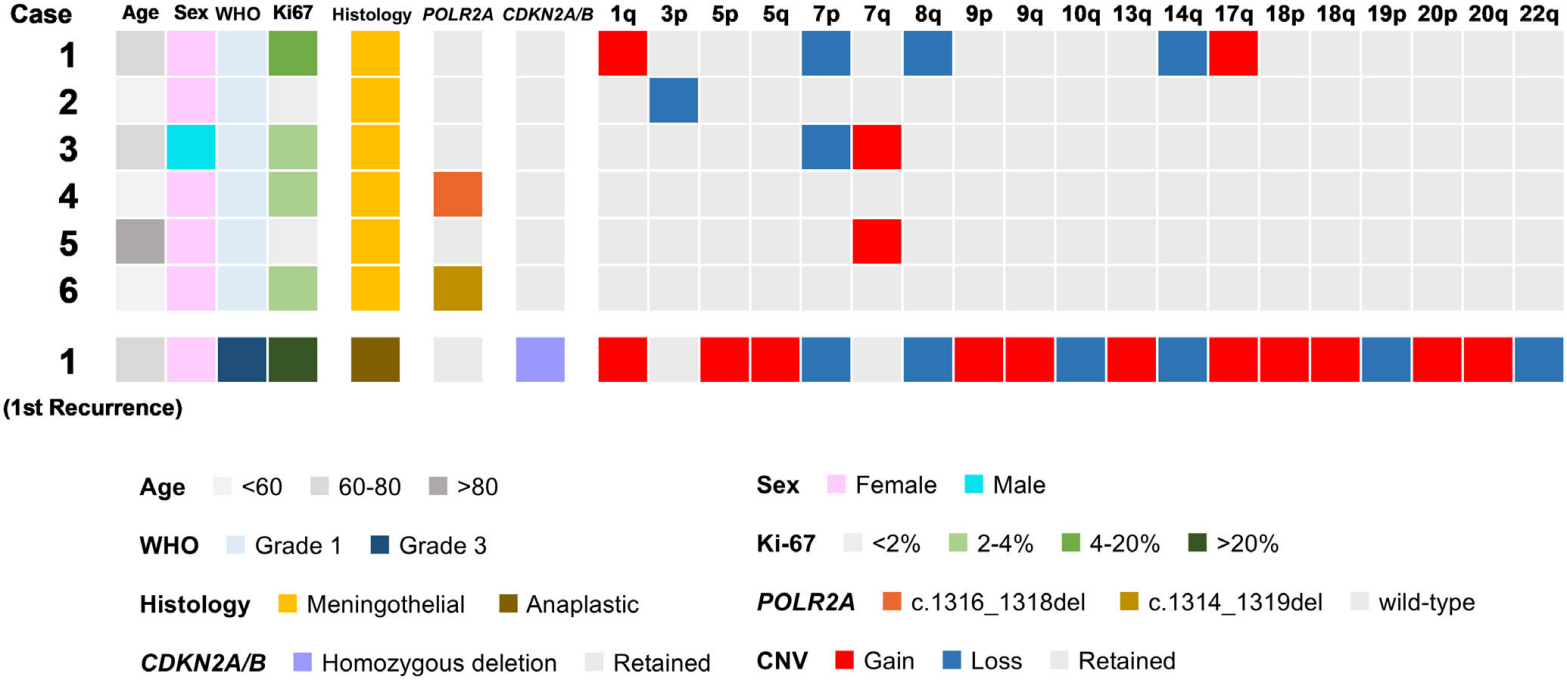
Mutational landscape. An oncoplot illustrating the mutational landscape of the six cases. CNV, copy number variations.

The genetic profiles of the primary tumors are summarized in Figure 2, and the mean overall sequencing depth as well as gene‐specific coverage for key meningioma‐associated genes are provided in Supporting Information S1. All binary alignment map files were manually inspected using integrative genomics viewer (IGV), and no *NF2* alterations were identified. *Case 1* is shown as a representative example of gene‐specific coverage in Figures S1 and S2. Two cases harbored *POLR2A* mutations (c.1316_1318del and c.1314_1319del). IGV snapshots of the *POLR2A*‐mutant cases are additionally presented as representative examples in Figure S3.

In *Case 1*, *BAP1* and *PBRM1* were retained despite the presence of partial rhabdoid features. Notably, *Case 1* nonetheless exhibited a telomeric deletion of chromosome 3p (Figure S4). However, this deletion spanned 3p26.3–3p25.1 and did not encompass the *BAP1* or *PBRM1* loci. While most cases harbored few arm‐level CNVs, *Case 1* exhibited multiple arm‐level CNVs already in the primary tumor, including 1q gain and 14q loss (Figure S5). *CDKN2A/B* was retained in the primary tumor of *Case 1*.

During follow‐up, five cases remained stable. In contrast, *Case 1* experienced multiple recurrences with progressive acquisition of CNVs, including 5p/q gain, 18p/q gain, 22q loss, and *CDKN2A/B* homozygous deletion. Given the history of fractionated radiotherapy, radiation‐induced chromothripsis was considered, but no evidence was identified. Of note, chromosome 22q was intact in the initial tumor but was lost at recurrence (Figures S5–S8).

### 3.3 Transcriptomic analyses

Integrated analysis of nine samples together with public datasets yielded a combined dataset comprising 964 samples.

**FIGURE 3:**
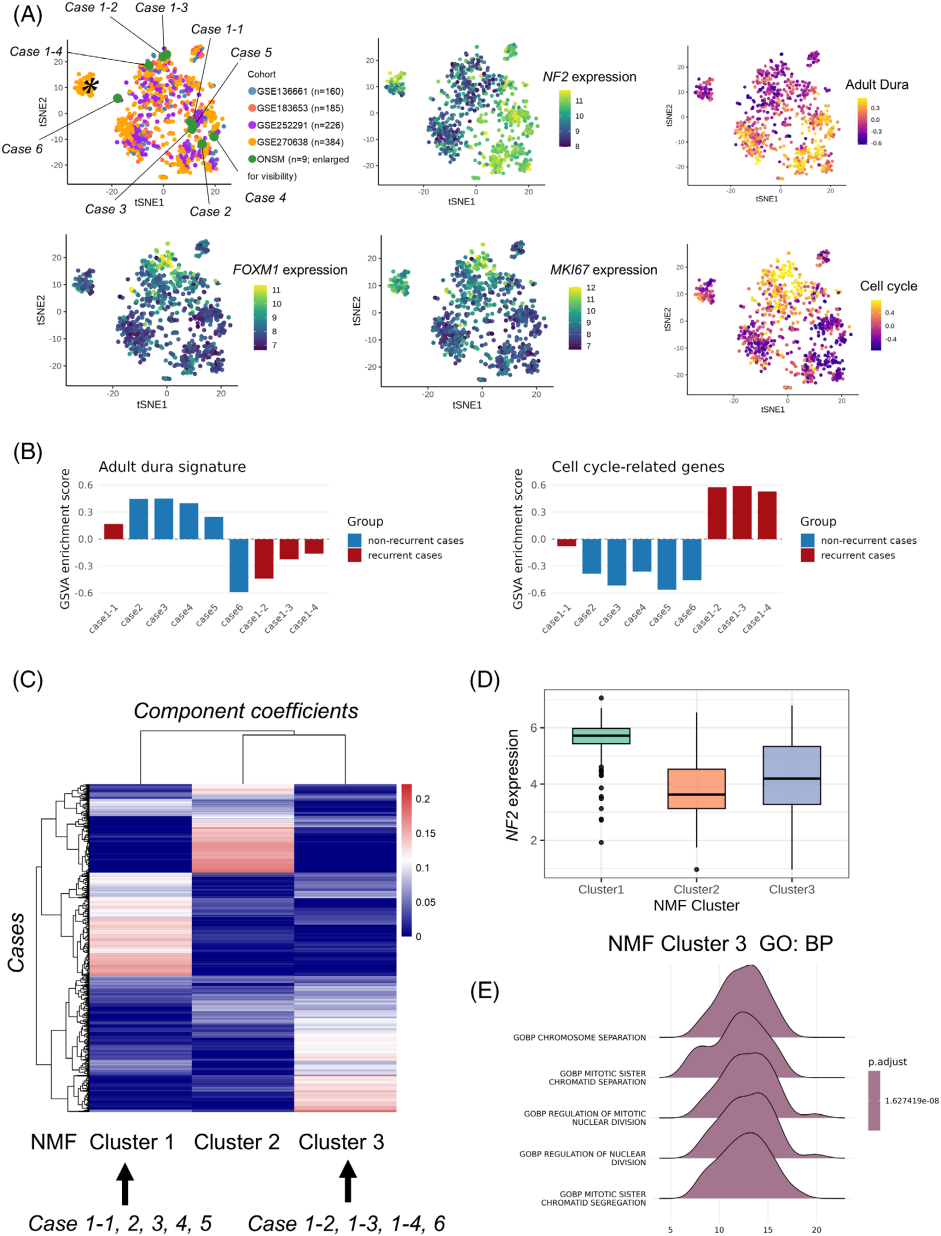
Transcriptomic landscape of optic nerve sheath meningioma (ONSM). *Case 1‐1* represents the primary tumor, whereas *Cases 1‐2*, *1‐3*, and *1‐4* correspond to recurrent lesions. (A) Visualization of the transcriptomic landscape of six ONSMs together with four public meningioma datasets using t‐distributed stochastic neighbor embedding (t‐SNE). Each ONSM is shown in relation to reference meningiomas. ONSM sample circles are enlarged for visibility. (B) Adult dural signature and cell cycle‐related gene expression are evaluated by Gene Set Variation Analysis (GSVA) for each ONSM. (C) Non‐negative matrix factorization (NMF) clustering was performed on log‐normalized, batch‐corrected transcript counts. *Case 1* at initial presentation and *Cases 2–5* grouped into the same NMF cluster, whereas *Case 1* at recurrence was classified into another cluster. (D) Cluster 1 showed relatively retained *NF2* expression. (E) Cluster 3 was characterized by hypermitotic activity, as indicated by enrichment of Gene Ontology Biological Process (GO:BP) terms related to cell‐cycle regulation.

First, t‐SNE was applied to batch‐corrected and normalized transcript counts, as described in the Methods section, and visualized in a two‐dimensional space (Figure 3A). A distinct cluster of 78 cases from GSE270638 (colored orange and marked with an asterisk) was identified. As these cases exclusively originated from a single institution (Princess Margaret Cancer Center) and included all samples from that cohort, this cluster was considered to represent a batch effect rather than a biologically meaningful group.

The transcriptional landscape revealed a clear gradient of *NF2* expression, the adult dural signature, and cell cycle‐related genes within the tumor cluster. ONSM did not form a distinct cluster separate from other meningiomas. The landscape indicated that *NF2* expression and the adult dural signature were largely preserved in most primary tumors but were progressively lost during tumor evolution, although *Case 6* did not align with that trend. In contrast, cell cycle‐related genes were upregulated in recurrent tumors compared with primary tumors (Figure 3B).

Next, NMF clustering was applied to all samples (*n* = 964). *Case 1* at initial presentation and *Cases 2–5* were grouped into the same NMF cluster, which was characterized by relatively preserved *NF2* expression. In contrast, *Case 1* at recurrence was assigned to the hypermitotic, proliferative NMF cluster with relatively lower *NF2* expression (Figure 3C–E).

**FIGURE 4:**
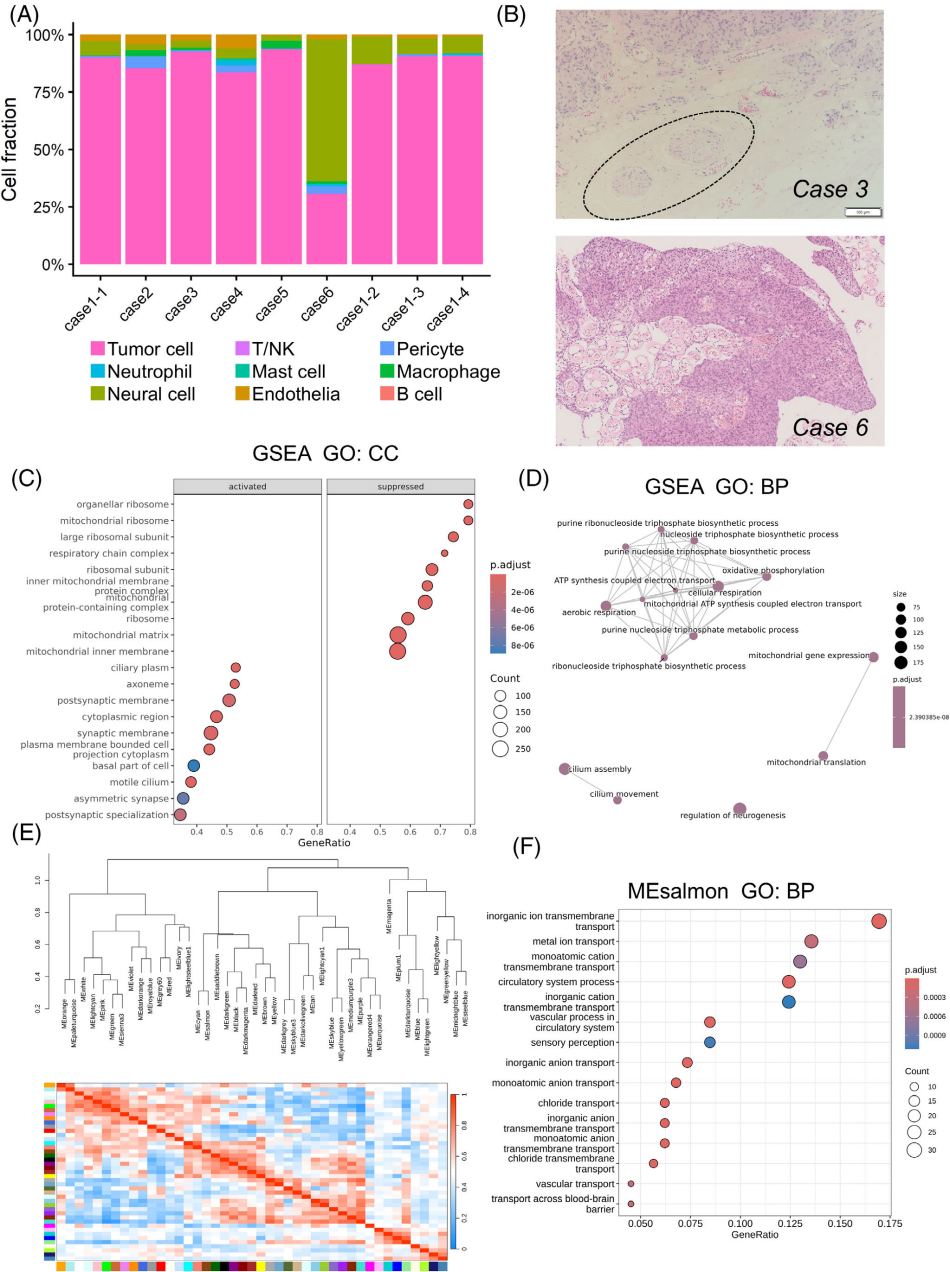
Characteristic transcriptomic features of optic nerve sheath meningioma (ONSM). (A) Cellular composition of each case inferred using BayesPrism. (B) Representative cases are presented, with neural tissue highlighted by a dotted circle. (C, D) Gene set enrichment analysis (GSEA) demonstrates significant enrichment of pathways associated with neuronal architecture. (E) Weighted gene co‐expression network analysis is performed on a combined cohort of primary and recurrent ONSM cases together with public meningioma samples (*n* = 964), and module eigengenes (MEs) are calculated. (F) Modules characteristic of primary ONSM show enrichment for neural‐related expression programs. GO:BP, Gene Ontology Biological Process; GO:CC, Gene Ontology Cellular Component; GSEA, gene set enrichment analysis.

Bulk deconvolution via BayesPrism was performed on nine institutional ONSM cases. Neural cells were inferred to be present across all tumor samples (Figure 4A). *Case 6* showed the highest inferred neural contribution. However, histopathological examination demonstrated that the resected specimen from *Case 6* did not contain any identifiable neural tissue. Similarly, no neural tissue was identified in *Cases 1*, *4*, or *5*. In contrast, neural structures were observed in the resected specimens from *Cases 2* and *3* (Figures 4B, S9, and S10), despite their lower inferred neural contribution by BayesPrism. In *Case 2*, large nerve bundles were identified within reactively fibrotic orbital fat and were considered consistent with ciliary nerves traversing the orbital fat [24]. In *Case 3*, fine nerve bundles accompanied by small vessels were observed within the optic nerve sheath itself, consistent with *nervi meningei* or *nervi nervorum* [37, 38].

Although the tissue sections used for histopathology and RNA sequencing were not strictly identical, they were derived from adjacent regions of the same tumor without intentional spatial separation, allowing the histologically evaluated sections to reasonably reflect the cellular composition of the RNA‐sequenced samples. Hence, BayesPrism appeared to be unable to fully recapitulate the histologically defined cellular composition and appeared to be influenced by neural‐associated transcriptional programs derived from non‐neural cells. Given this limitation, transcriptomic analyses were performed independently of cell type deconvolution.

First, we identified DEGs (Figure S11) and performed GSEA by comparing six primary ONSM cases with 955 publicly available meningioma cases to delineate transcriptomic programs characteristic of ONSM. Recurrent cases were excluded from this analysis because they had undergone multiple courses of radiotherapy, as shown in Figure 1C, and were therefore considered biologically distinct from primary ONSM. This analysis included a total of 961 cases. GSEA consistently identified enrichment of neuronal architecture‐related processes, including axons, synapses, microtubules, and dynein‐associated complexes (Figure 4C,D), highlighting a transcriptomic landscape of ONSM closely linked to the optic nerve microenvironment.

To reduce potential confounding from neural tissue admixture, *Cases 2* and *3* were excluded, and the analyses were repeated as a sensitivity analysis, yielding a cohort of 959 cases. Enrichment of neural niche‐associated programs was preserved in the sensitivity analysis (Figure S12).

To further validate these findings, WGCNA was performed across primary and recurrent ONSM cases together with public meningioma samples, in a combined cohort of 964 cases. This analysis identified well‐defined and reproducible co‐expression modules, indicating robust network construction across the integrated dataset (Figures 4E and S13A,B). Modules characteristic of primary ONSM showed enrichment for neural‐related expression programs (Figure 4F), whereas recurrent cases exhibited prominent metabolic and cell cycle‐related programs, supporting the biological validity of the network analysis (Figure 13C).

## 4 DISCUSSIONS

The genetic landscape of ONSM has long remained under the veil because of the rarity of the disease and the limited availability of tumor specimens, as surgical resection is generally reserved as a last resort [4], whereas radiotherapy remains the main therapeutic option [39]. Although the number of analyzed samples was limited, we found that ONSM is characterized by the absence of *NF2* alterations and a paucity of CNVs, a molecular pattern closely resembling that of other skull base meningiomas. ONSM exhibits a transcriptomic profile that reflects its anatomical singularity and inextricable association with the neuronal architecture. Although ONSMs are generally indolent and non‐life‐threatening, malignant evolution driven by progressive CNV accumulation can occur in rare cases.

Landmark studies have demonstrated that *NF2*‐intact meningiomas harbor mutually exclusive somatic mutations in *TRAF7*, *KLF4*, AK*T1*, *SMO*, and *POLR2A* [40, 41, 42]. These tumors typically exhibit few CNVs, while cases lacking these mutations occasionally display chromosomal gains [9, 43]. Our results were generally in line with the prevailing concept, as all tumors in our cohort were *NF2*‐intact and, except for *Case 1*, showed minimal CNV changes. Notably, the spectrum of genetic alterations is tightly linked to anatomical location [27, 44, 45, 46, 47], and *NF2*‐intact tumors preferentially arise from the skull base [9, 48, 49, 50]. Because the orbit is closely aligned with the skull base, it is reasonable that ONSM rarely exhibits *NF2* alterations. In addition, the frequent occurrence of *POLR2A* mutations in tuberculum sellae meningiomas [41, 49] may explain the enrichment of *POLR2A* alterations in ONSM, as the optic canal and orbit are in close anatomical continuity with the tuberculum sella.

However, the interpretation of *POLR2A* alterations across established classification systems warrants caution. While *POLR2A*‐mutant tumors were incorporated within benign *NF2* wild‐type categories in the Toronto multi‐omic framework [9], they were variably distributed or split across multiple subclasses in other systems, including the DKFZ methylation classifier [15], and the Baylor multi‐omic framework [12]. This inter‐study discrepancy suggests that the presence of *POLR2A* mutations alone is insufficient to unambiguously assign tumors to a single molecular subgroup. Notably, a similar phenomenon was observed in our own transcriptomic analysis: although the majority of ONSM primary samples clustered within the *NF2* wild‐type group, one *POLR2A*‐mutant case (*Case 6*) segregated into a distinct cluster, underscoring the intrinsic heterogeneity of *POLR2A*‐altered meningiomas.

Although genome‐wide methylation profiling was not performed in the present study, we sought to address this limitation by applying unsupervised transcriptomic clustering. Following the transcriptome‐based clustering of meningiomas reported by the Baylor group in 2019 [13], which identified molecularly distinct groups using gene expression profiles without reliance on methylation data, we performed NMF analysis in our cohort. In this framework, all but one ONSM primary cases were grouped with *NF2* wild‐type‐associated transcriptomic clusters (Figure 3D,E). Collectively, these findings support the conceptual alignment of ONSM with benign *NF2*‐intact, skull base meningiomas, while also highlighting the molecular heterogeneity observed among *POLR2A*‐mutant tumors.

While ONSM is generally considered non‐life‐threatening [3, 6, 22, 23], we encountered an exceptional case with an aggressive clinical course. The tumor already harbored multiple CNVs at its initial presentation, including high‐risk alterations [9, 10, 12, 13, 14, 15, 51] such as 1q gain and 14q loss. Both are well known for their close association with poor prognosis [16, 52, 53, 54, 55]. During recurrence, it acquired additional aberrations, most notably *CDKN2A/B* homozygous deletion—an alteration well known for its malignant implications [8, 14, 56, 57]. Our findings suggest that ONSM can rarely undergo malignant transformation through mechanisms that resemble those driving tumor evolution in other intracranial meningiomas. Given that this patient had previously received fractionated radiotherapy, we considered the possibility that treatment‐related genomic instability, such as chromothripsis‐like rearrangements [58], might have contributed to tumor progression. However, no such patterns were detected in our analysis. Importantly, these rare instances may harbor high‐risk CNVs already at diagnosis, even in the absence of canonical driver mutations.

An intriguing observation is that the recurrent case already exhibited rhabdoid morphology at the initial surgery, despite the absence of *BAP1* or *PBRM1* alterations. These rhabdoid features were partially retained across subsequent recurrences. A recent report of a rhabdoid meningioma harboring a *BAP1* alteration in ONSM has drawn attention to the potential biological relevance of rhabdoid features in this anatomical context [25]. While definitive conclusions cannot be drawn from these limited cases, the available observations suggest that rhabdoid differentiation can be observed in ONSM and may be associated with an unfavorable clinical outcome. Also, despite the absence of *BAP1* alterations, our case exhibited rhabdoid morphology, underscoring that the relationship between *BAP1* status and rhabdoid differentiation is not deterministic, although a general association has been recognized [59, 60, 61].

The acquisition of chromosome 22q loss at a mid‐stage of the clinical course of *Case 1* is uncommon and has not been well characterized [62]. Concordant evidence from CNVkit‐based whole‐exome analysis, RNA sequencing‐based CNV inference, and *NF2* expression supports the interpretation that chromosome 22q loss was acquired during tumor progression. While low‐frequency subclonal loss at initial presentation cannot be completely ruled out, the consistent distinction between intact and lost states across multiple independent modalities argues against technical threshold effects. Although 22q loss is widely regarded as an early tumorigenic event [63, 64, 65, 66], our case, together with a sporadic prior report [62], suggests that 22q loss may represent a later, evolutionarily acquired alteration rather than an initiating event. Moreover, despite *NF2*‐intact and 22q‐loss tumors being considered distinct molecular entities in current multi‐omic classifications [9, 10, 12, 13, 15], our longitudinal observation raises the possibility that a subset of *NF2*‐intact tumors may acquire 22q loss over time, accompanied by increasing chromosomal complexity.

Transcriptomic profiling revealed enrichment of gene sets associated with the optic apparatus, neuronal architecture, and axonal transport. To assess the contribution of neural components, cellular composition was inferred using BayesPrism. However, the inferred results were discordant with histopathological evaluation. No neural tissue was identified in *Cases 1*, *4*, *5*, or *6*, whereas neural tissue was present in *Cases 2* and *3*, represented by ciliary nerves in *Case 2* and *nervi meningei* or *nervi nervorum* in *Case 3*.

These findings have several implications. First, BayesPrism was unable to fully recapitulate histologically defined cellular composition and appeared to be influenced by neural‐associated transcriptional programs derived from non‐neural cells, indicating that higher resolution approaches such as single cell or spatial transcriptomic analyses will be required for definitive dissection [67, 68, 69]. Second, surgical specimens of ONSM may contain multiple distinct neural components, including the optic nerve itself, ciliary nerves, and *nervi meningei* or *nervi nervorum*, with important implications for pathological and transcriptomic interpretation. Third, despite the absence of microscopically identifiable neural tissue, *Case 6* exhibited an indistinct tumor optic nerve interface on preoperative imaging, suggesting that close tumor nerve interaction at the macroscopic level may induce neural‐associated gene expression programs without direct neural cell invasion.

Another issue worth discussing is that, while GSEA indicated enrichment of neuronal and glial pathways, no individual genes characteristic of these cell types reached statistical significance in the differential expression analysis. These findings suggest two possibilities: first, that ONSM harbors a coordinated pathway‐level transcriptional signature rather than discrete gene‐level alterations; and second, that the limited number of cases may have reduced the statistical power to detect individual gene‐level changes.

Nevertheless, our data raise the possibility that ONSM harbors transcriptomic features that reflect its unique intimacy with adjacent neural structures. Unlike other cranial nerves, which are myelinated by Schwann cells and enveloped by a perineurium, the optic nerve is myelinated by oligodendrocytes and ensheathed in the meninges, reflecting its nature as a direct extension of the central nervous system [22, 70]. Accordingly, no other intracranial meningioma maintains an equally profound association with the cranial nerves, which represents a truly unique anatomical context. Among meningiomas, ONSM is unique in being named after a cranial nerve. This identity may underlie a dynamic dialog between the tumor and its neural milieu, quietly shaping the tumorigenesis and evolutionary trajectory of ONSM.

## 5 CONCLUSIONS

ONSM is typically *NF2*‐intact and generally exhibits few CNVs, resembling other skull base meningiomas. In contrast, the transcriptomic profile of ONSM reflects its close association with the optic nerve microenvironment. Although usually indolent, certain cases may harbor high‐risk CNVs at diagnosis and evolve into an aggressive disease through further accumulation of CNV burdens.

## AUTHOR CONTRIBUTIONS

*Conceptualization*: Daisuke Sato and Satoru Miyawaki. *Data curation*: Daisuke Sato, Satoru Miyawaki, Kenta Ohara, Yudai Hirano, and Motoyuki Umekawa. *Formal analysis*: Daisuke Sato. *Resources*: Satoru Miyawaki and Nobuhito Saito. *Writing original draft*: Daisuke Sato. *Writing review and editing*: Satoru Miyawaki, Yu Sakai, Kenta Ohara, Yu Teranishi, Yudai Hirano, Motoyuki Umekawa, Takahiro Tsuchiya, Shotaro Ogawa, So Hirata, Hiroki Hongo, Hideaki Ono, Daisuke Komura, Tetsuo Ushiku, Shumpei Ishikawa, and Nobuhito Saito. *Visualization*: Daisuke Sato. *Supervision*: Satoru Miyawaki and Nobuhito Saito. *Funding acquisition*: Daisuke Sato, Satoru Miyawaki, and Nobuhito Saito.

## CONFLICT OF INTEREST STATEMENT

The authors report no conflicts of interest concerning the materials or methods used in this study or the findings specified in this paper.

## ETHICS STATEMENT

The study was approved by the Institutional Ethics Committee (G10028), and written informed consent was obtained from all participants.

## Supporting information


**Data S1.** Supporting Information.

Table S1.

## Data Availability

One case (*Case 1*) was included in a previously published multi‐omics study analyzing paired pre‐ and post‐recurrence meningioma specimens to delineate molecular alterations associated with malignant transformation [26]. The corresponding sequencing data are publicly available through the National Bioscience Database Center (NBDC) under accession numbers JGAS000820 and JGAS000299. De‐identified genomic and transcriptomic data for the remaining cases (*Cases 2–6*), newly generated in this study, are not publicly available because public data deposition was not permitted under the original informed consent and institutional ethics approval. These data are available from the corresponding author upon reasonable request and subject to approval by the institutional ethics committee.
