# Supplementary material for: Optic nerve sheath meningioma exhibits neural niche‐associated transcriptomic features and rare copy number variation‐linked evolution
Source: Brain Pathol. 2026 Feb 1;36(4):e70078. doi: 10.1111/bpa.70078 (PMC13239774; doi:10.1111/bpa.70078)
Supplement: Supplementary file 1 — Data S1. Supporting Information. [file BPA-36-e70078-s001.docx]

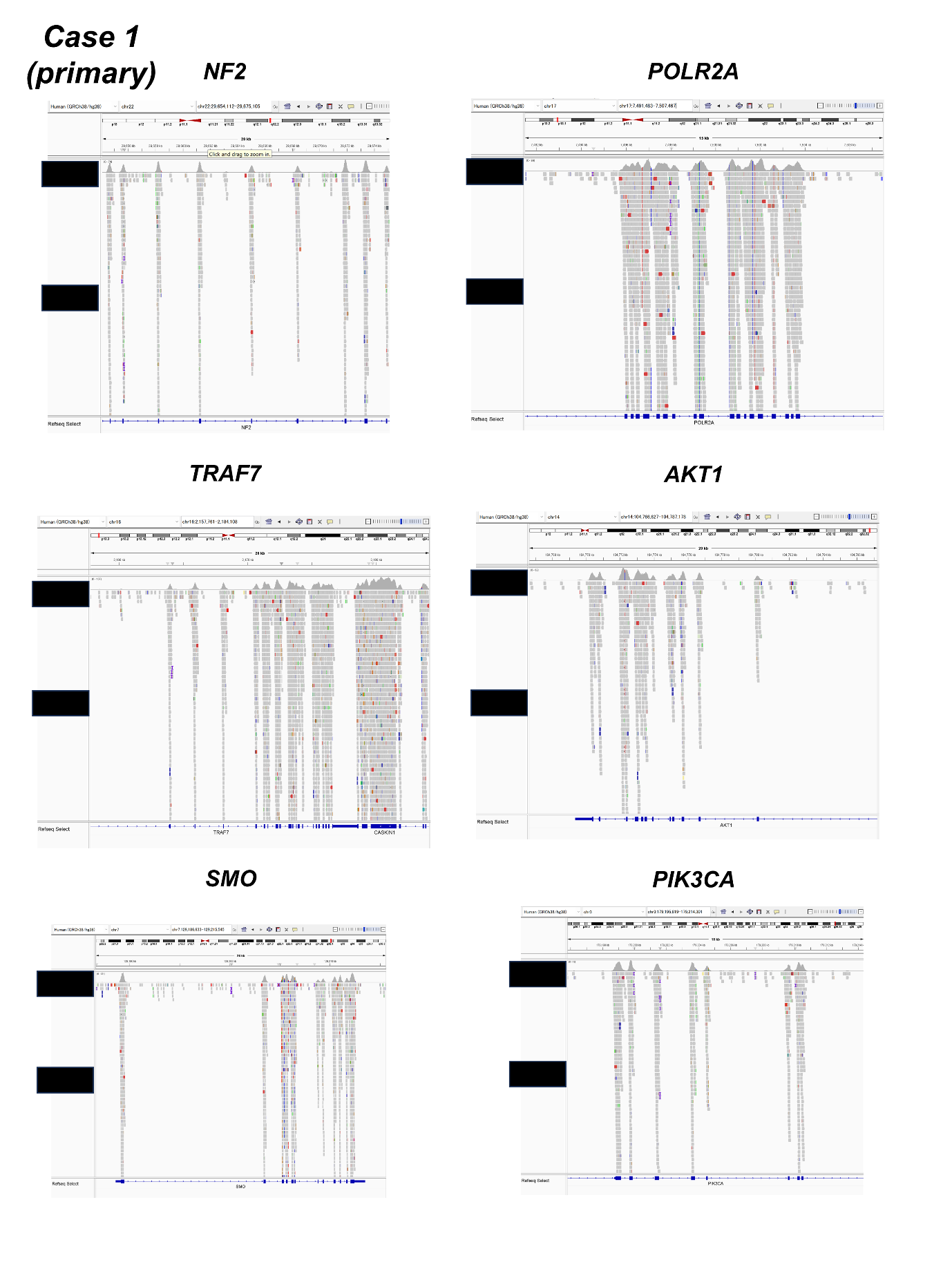


**Supplementary Figure 1.** IGV snapshots of Case 1 (part 1).
Read alignments for key genes are shown, demonstrating sufficient sequencing coverage.


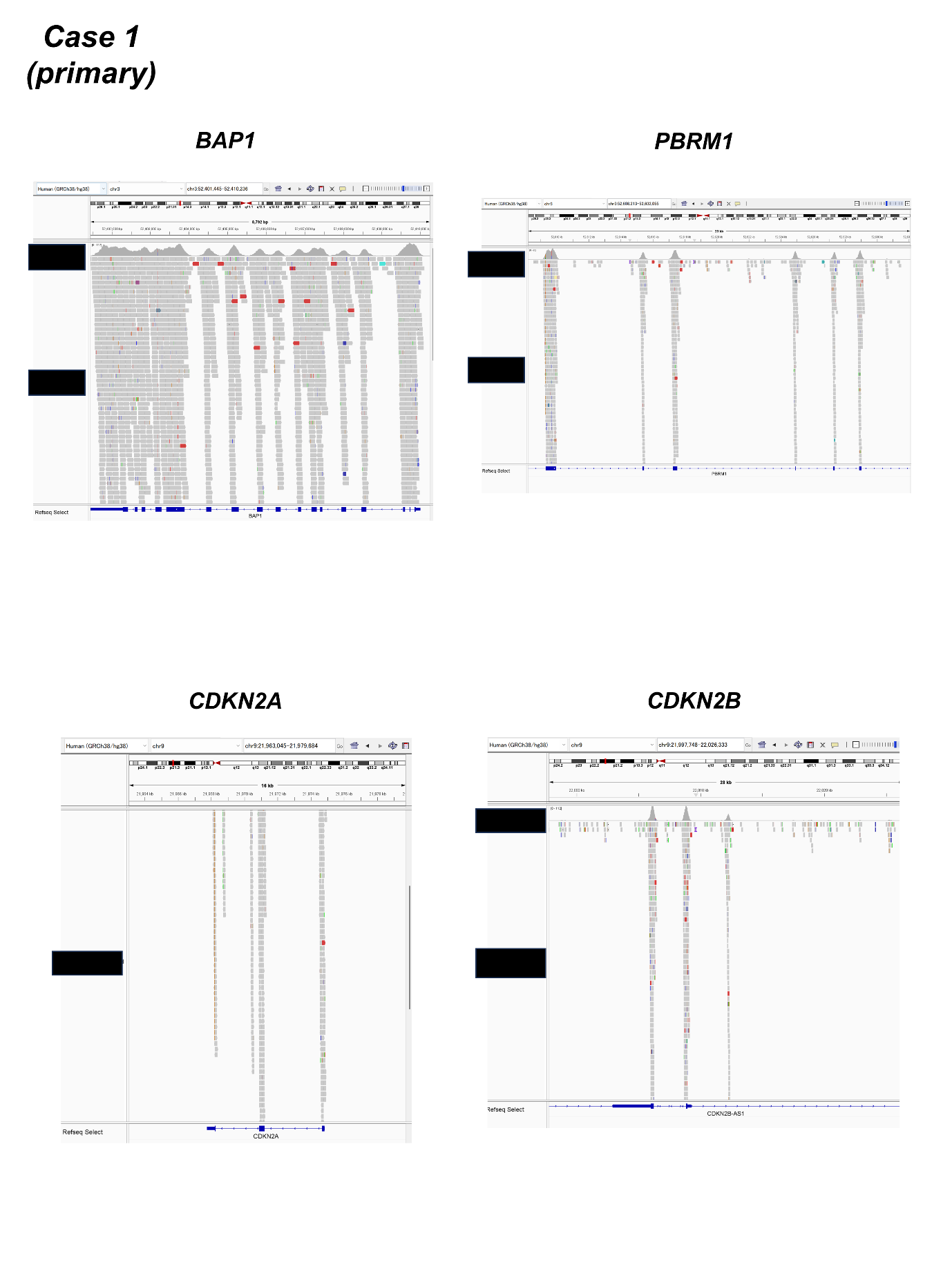


**Supplementary Figure 2.** IGV snapshots of Case 1 (part 2).
Read alignments for *BAP1*, *PBRM1*, and *CDKN2A/B* are shown, demonstrating sufficient sequencing coverage.


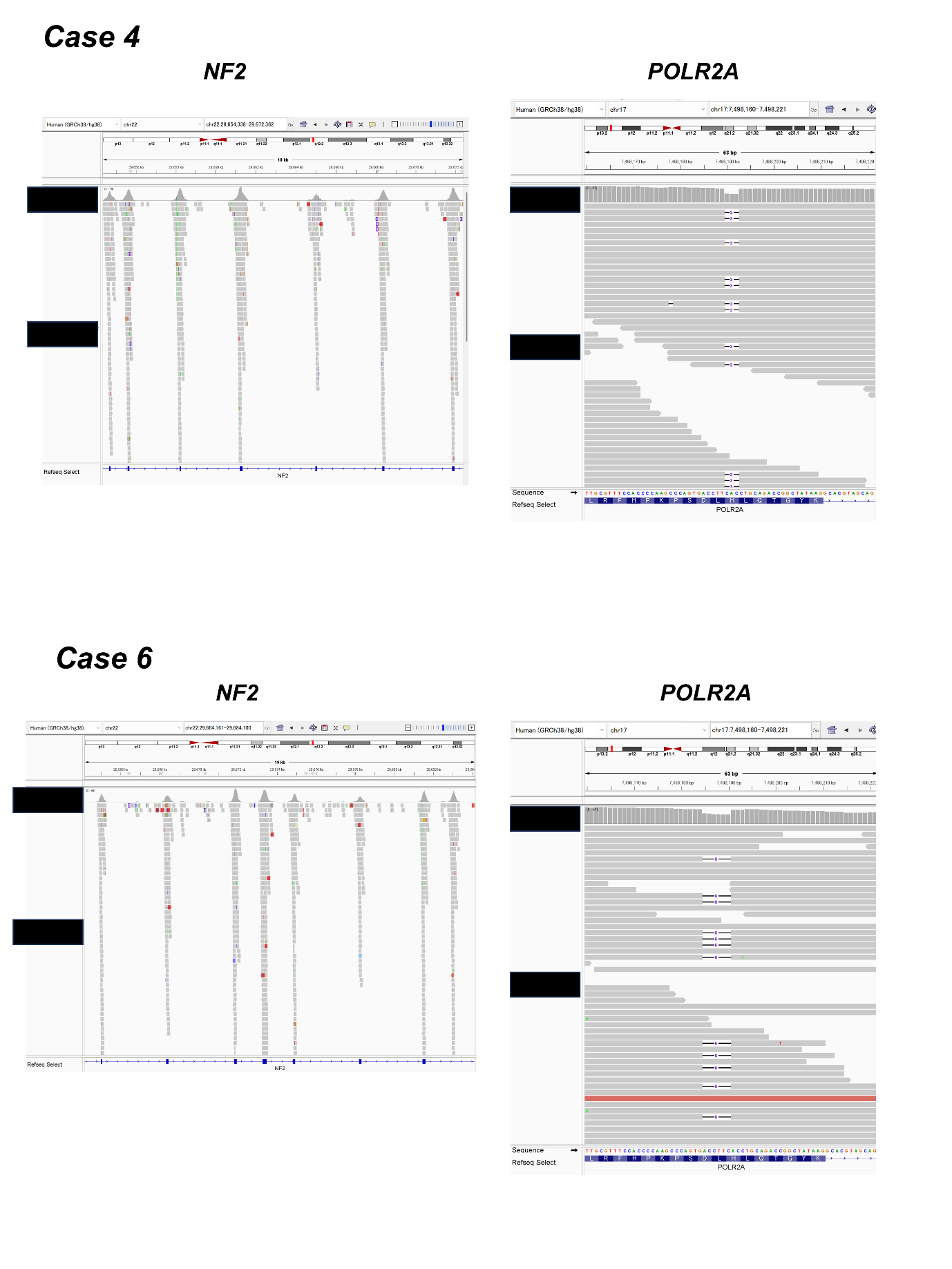


**Supplementary Figure 3.** IGV snapshots of *POLR2A*-mutant cases.
Representative IGV snapshots showing read alignments for *NF2* and *POLR2A* in *POLR2A*-mutant tumors are presented.


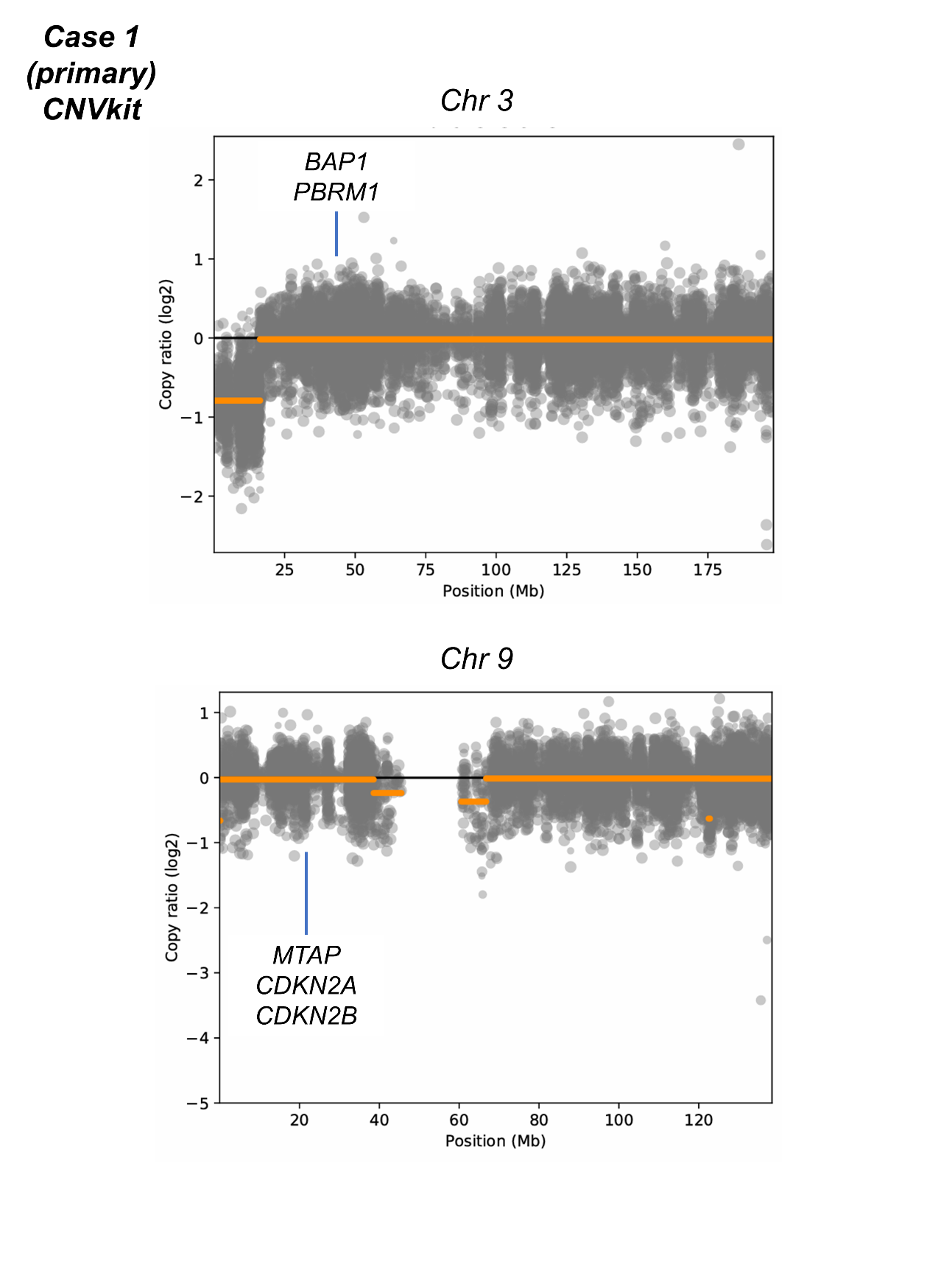


**Supplementary Figure 4.** CNVkit copy number profiles of the primary tumor from *Case 1* across chromosomes 3 and 9.
The loci encompassing *BAP1* and *PBRM1* (chromosome 3), as well as *CDKN2A/B* and *MTAP* (chromosome 9), showed no evidence of copy number loss.


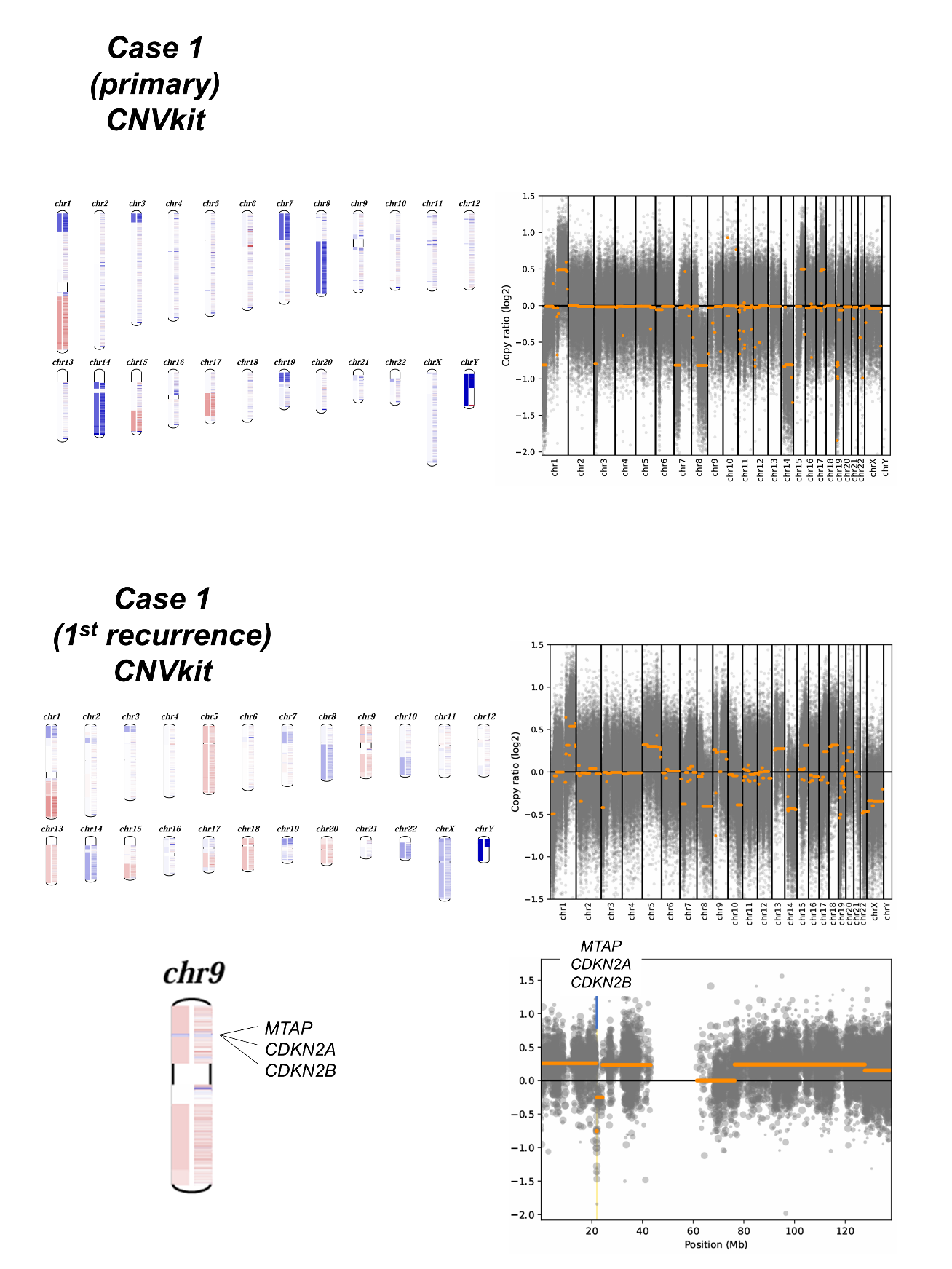


**Supplementary Figure 5.** CNVkit copy number profiles of the primary tumor and first recurrent tumor from *Case 1*.
IGV analysis revealed the acquisition of multiple copy number alterations at the first recurrence, including loss of *CDKN2A/B* and chromosome 22q.


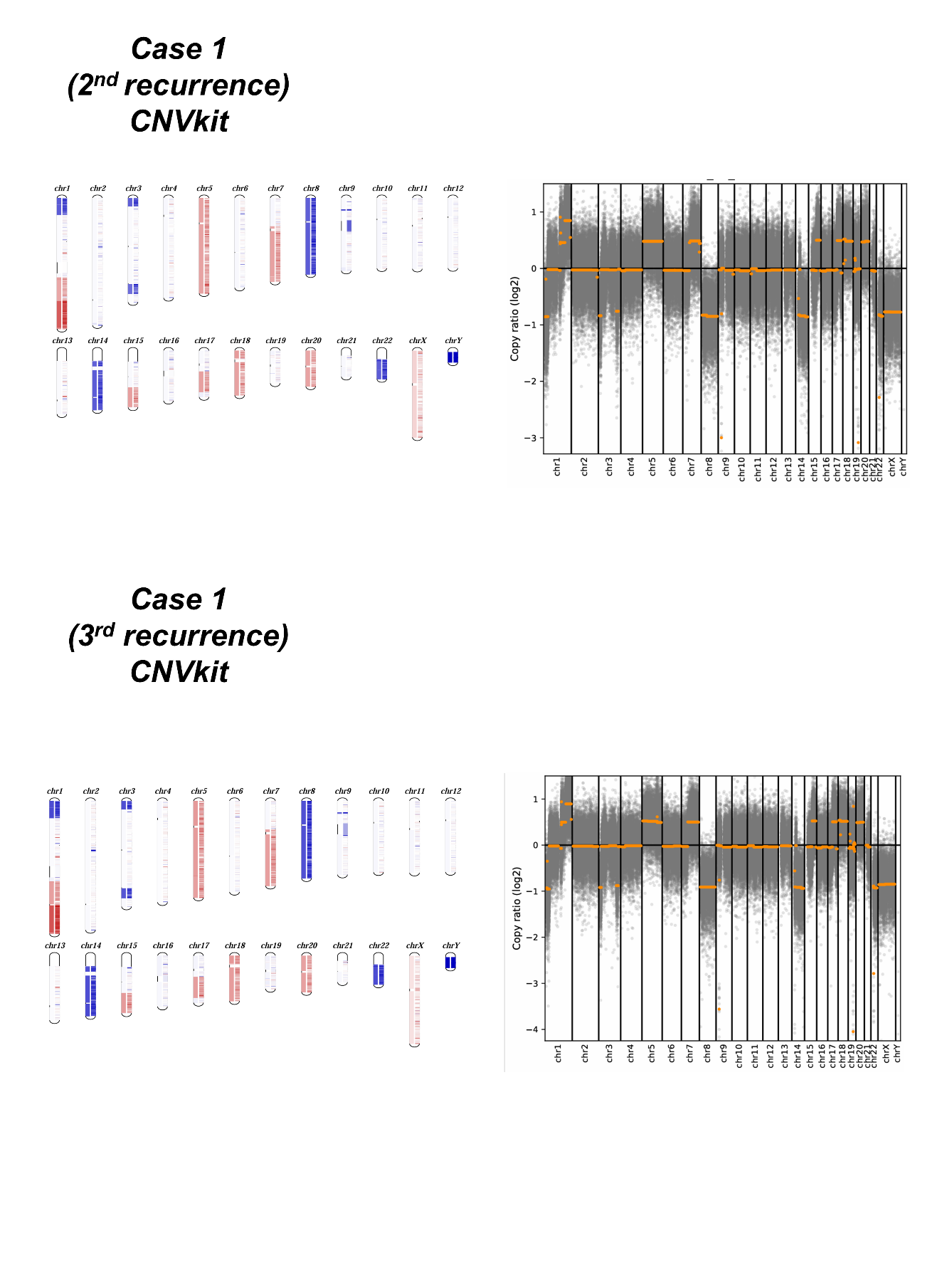


**Supplementary Figure 6.** CNVkit copy number profiles of the second tumor and third recurrent tumor from *Case 1*.
The two tumors demonstrated highly concordant copy number alteration profiles, characterized by gains of 1q, 5p/q, and 20p/q, and losses of 8p/q, 14q, 18p/q, and 22q.


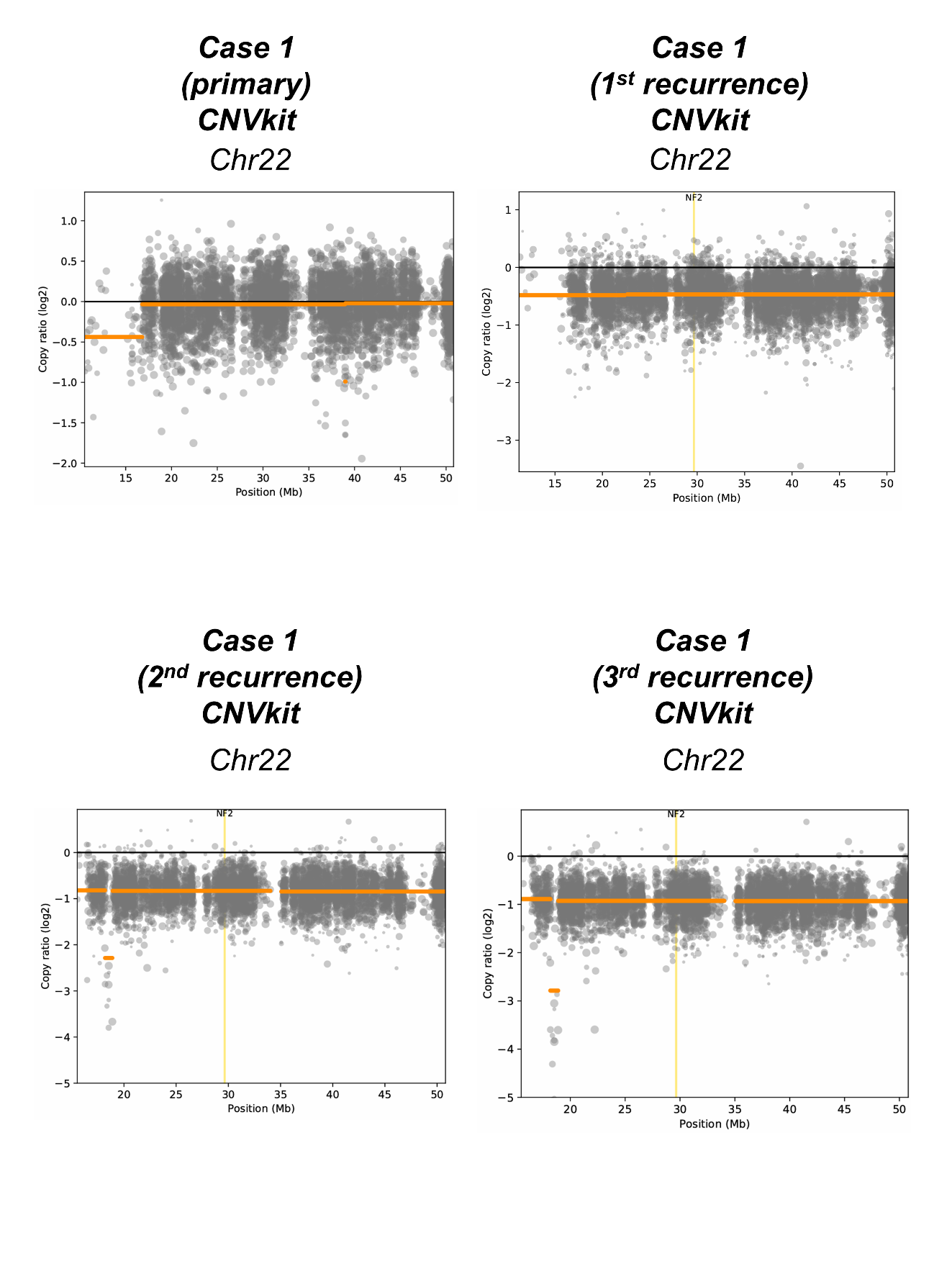


**Supplementary Figure 7.** Comparison of chromosome 22 in the primary and recurrent tumors from *Case 1*.
Chromosome 22q was intact in the initial tumor but was lost in all recurrent tumors.


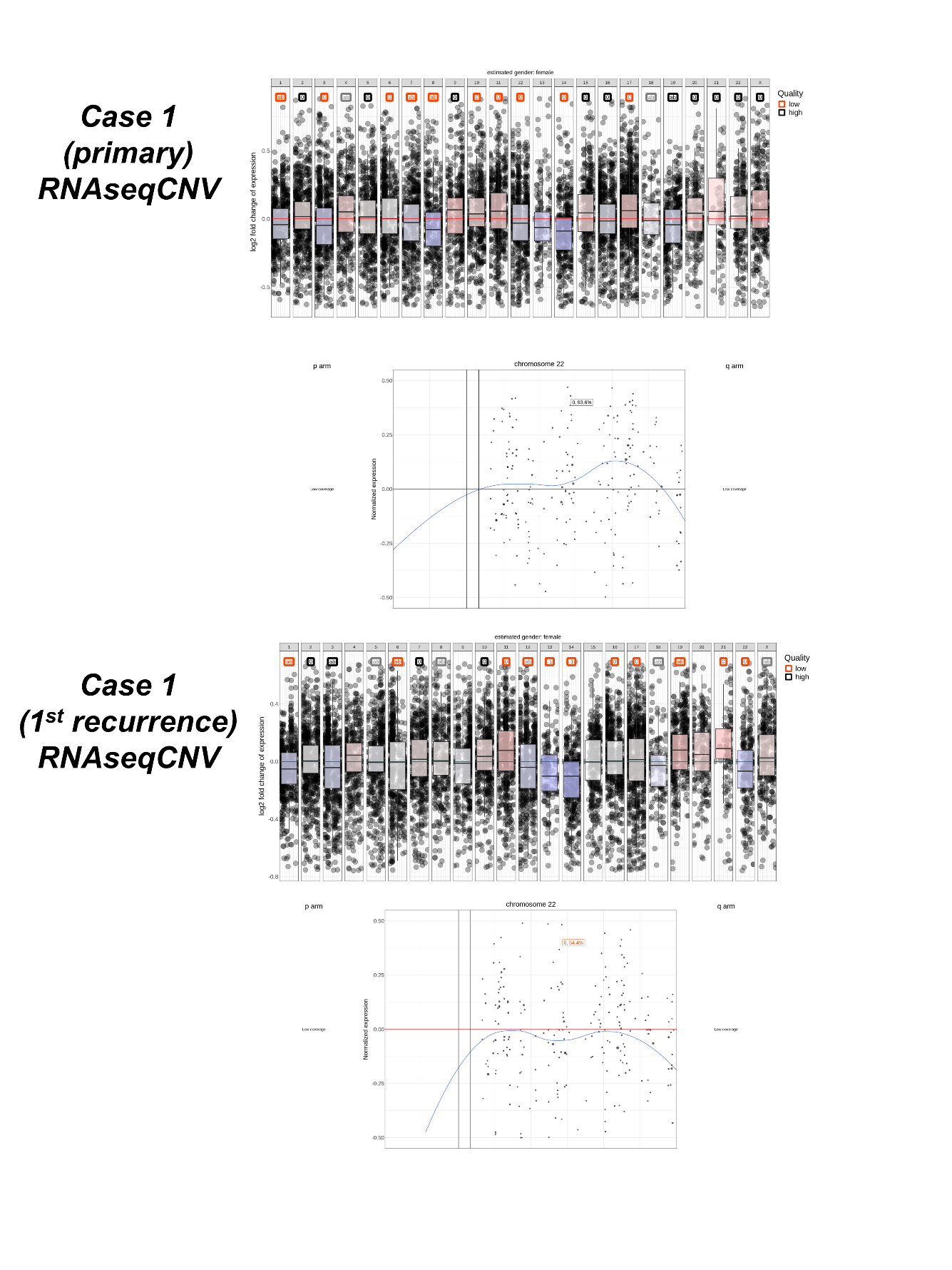


**Supplementary Figure 8.** RNAseqCNV copy number analysis of *Case 1*.
RNAseqCNV demonstrated an intact chromosome 22q in the primary tumor and loss of chromosome 22q in the recurrent tumor.


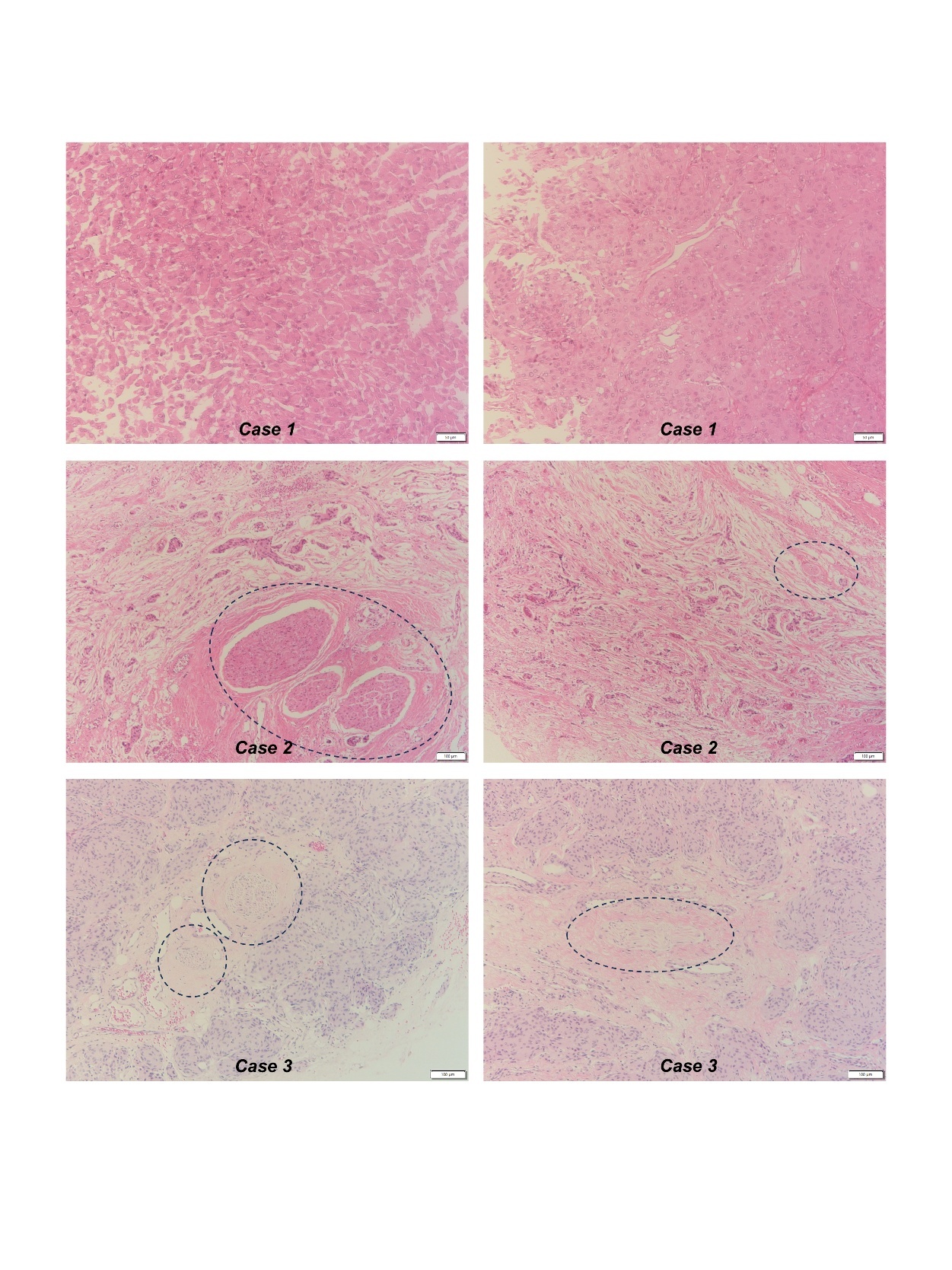


**Supplementary Figure 9.** Representative histopathological images of *Cases 1–3*.
*Case 1* shows focal rhabdoid features, although no identifiable neural components are present. *Case 2* demonstrates tumor infiltration into fibrous tissue with reactive fibrosis, accompanied by small and large nerve bundles (dotted circle), presumed to represent ciliary nerves traversing the orbital fat. *Case 3* contains neural structures (dotted circle) consistent with *nervi meningei* or *nervi nervorum* within the optic nerve sheath.


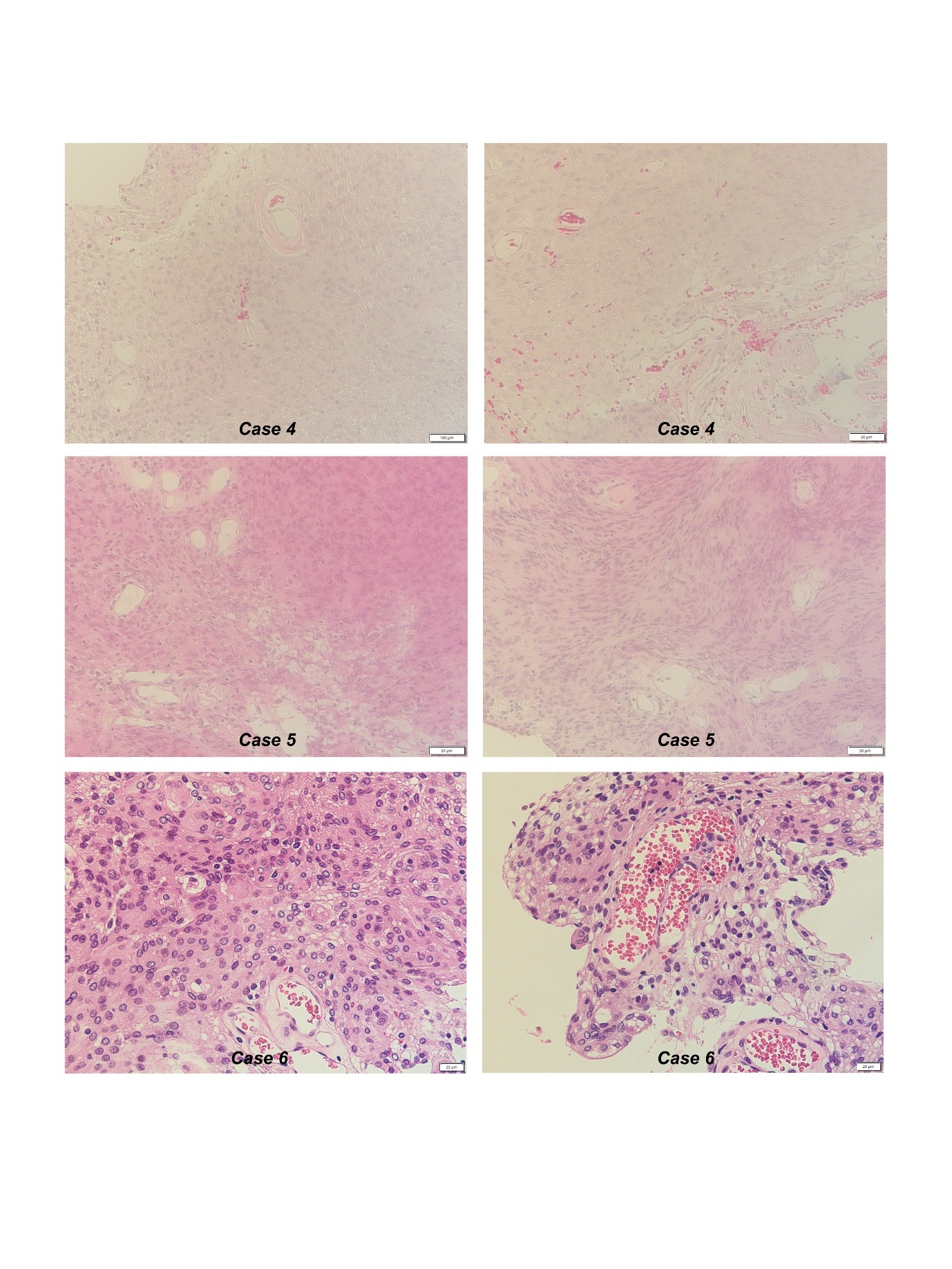


**Supplementary Figure 10.** Representative histopathological images of *Cases 4–6*.
No identifiable neural tissue was observed in *Cases 4–6*.


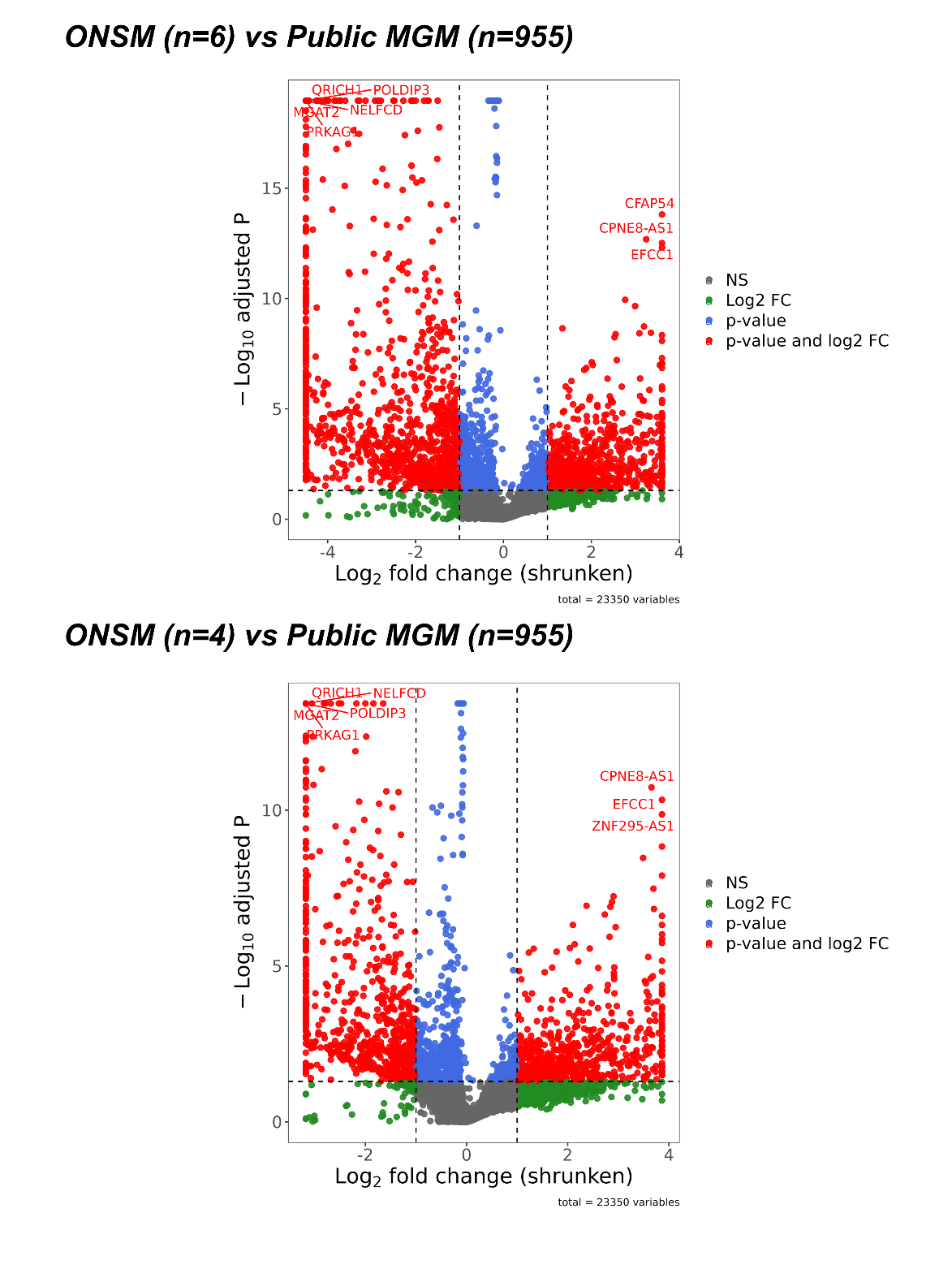


**Supplementary Figure 11.** Volcano plots comparing ONSM with public meningioma samples.
Differential expression analyses were performed comparing ONSM cases (n = 6) versus public meningiomas (n = 955), as well as a restricted comparison excluding neural tissue–contaminated cases (ONSM n = 4 versus public meningiomas n = 955). Both analyses identified multiple differentially expressed genes; however, these genes were not, in isolation, directly indicative of a neural niche–associated transcriptional program.

meningioma, MGM


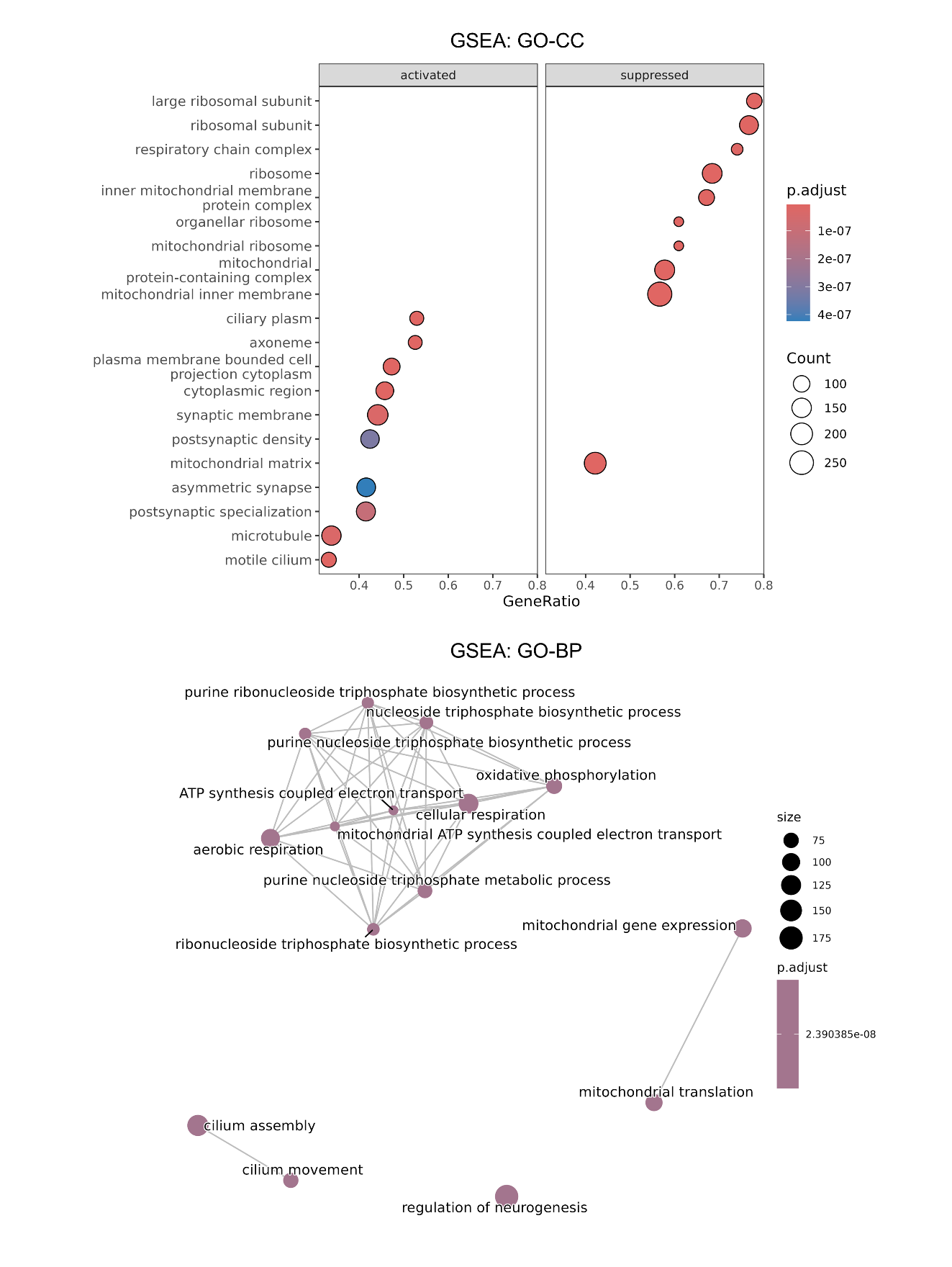


**Supplementary Figure 12.** Gene set enrichment analysis (GSEA) of ONSM (n = 4) versus public meningioma (n = 955) samples.
As a sensitivity analysis excluding cases with neural tissue admixture, GSEA continued to demonstrate significant enrichment of neural-related pathways.

gene set enrichment analysis, GSEA; meningioma, MGM


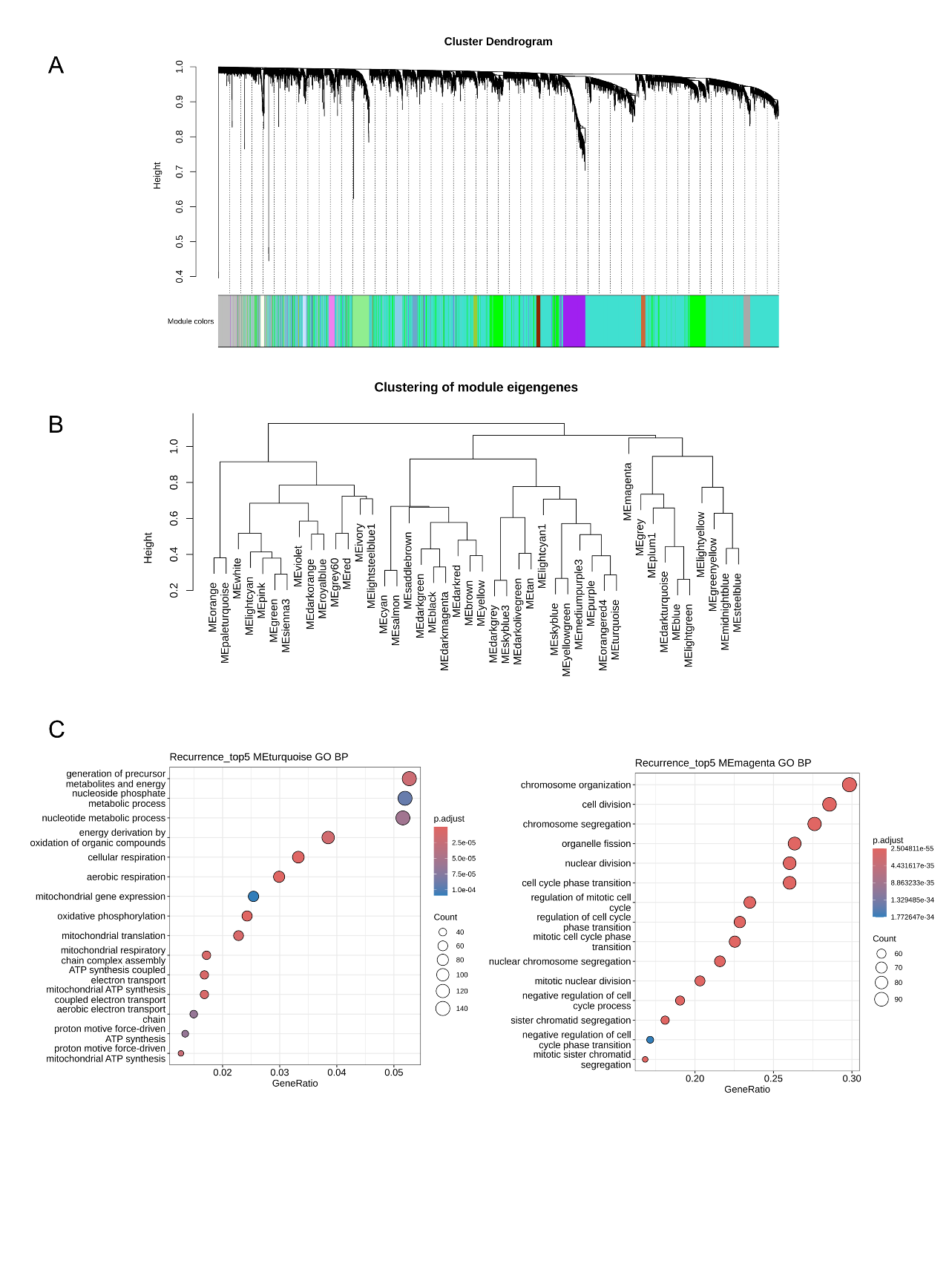


**Supplementary Figure 13.** WGCNA of ONSM together with public meningioma samples.
**A–B**, Weighted gene co-expression network analysis (WGCNA) identified multiple co-expression modules, summarized by their module eigengenes (MEs). **C**, Recurrent cases showed enrichment of hypermetabolic and proliferative module eigengenes, as evaluated by Gene Ontology biological process (GO:BP) analysis.

Gene Ontology biological process, GO:BP; module eigengenes, MEs; weighted gene co-expression network analysis, WGCNA
